# Raman Spectroscopy Study of Curvature-Mediated Lipid Packing and Sorting in Single Lipid Vesicles

**DOI:** 10.1016/j.bpj.2019.09.020

**Published:** 2019-09-20

**Authors:** Liam Collard, Faris Sinjab, Ioan Notingher

**Affiliations:** 1 School of Physics and Astronomy, University of Nottingham, University Park, Nottingham, United Kingdom

## Abstract

Cellular plasma membrane deformability and stability is important in a range of biological processes. Changes in local curvature of the membrane affect the lateral movement of lipids, affecting the biophysical properties of the membrane. An integrated holographic optical tweezers and Raman microscope was used to investigate the effect of curvature gradients induced by optically stretching individual giant unilamellar vesicles (GUVs) on lipid packing and lateral segregation of cholesterol in the bilayer. The spatially resolved Raman analysis enabled detection of induced phase separation and changes in lipid ordering in individual GUVs. Using deuterated cholesterol, the changes in lipid ordering and phase separation were linked to lateral sorting of cholesterol in the stretched GUVs. Stretching the GUVs in the range of elongation factors 1–1.3 led to an overall decrease in cholesterol concentration at the edges compared to the center of stretched GUVs. The Raman spectroscopy results were consistent with a model of the bilayer accounting for cholesterol sorting in both bilayer leaflets, with a compositional asymmetry of 0.63 ± 0.04 in favor of the outer leaflet. The results demonstrate the potential of the integrated holographic optical tweezers-Raman technique to induce deformations to individual lipid vesicles and to simultaneously provide quantitative and spatially resolved molecular information. Future studies can extend to include more realistic models of cell membranes and potentially live cells.

## Significance

We report a noninvasive technique to induce curvature gradients in a single-cell-sized lipid vesicle and simultaneously measure phospholipid chain packing, lateral distribution, and interleaflet asymmetry of cholesterol. Membrane curvature is known to play a role in the distribution of lipids and links with many biological processes. Fluorescence studies showed that curvature gradients can induce phase separation in a lipid vesicle; however, these studies are reliant on fluorescent labels that may alter the interactions governing lipid phase behavior. Here, we apply Raman spectroscopy to measure differences in cholesterol content in an optically stretched giant vesicle. By comparing the experimental observations with a mathematical model, we are able to predict the distribution of cholesterol in the two leaflets of the bilayer.

## Introduction

The high deformability of plasma membrane is essential in many cellular processes, including cell division, motility, and endo- and exocytosis. The underlying structure of the plasma membrane consists of a lipid bilayer, containing phospholipids, glycolipids, and sterols. The bilayer is laterally heterogeneous, featuring regions of disordered and ordered domains with different molecular compositions, leading to different biophysical properties (1). Bending of the membrane leads to curvature gradients across the bilayer, affecting lipid ordering and spatial distribution. The curvature gradients can induce sorting of individual lipid molecules or lateral movement of whole microdomains (2, 3). However, the way in which these microdomains form and the composition and asymmetry between the bilayer leaflets are still not understood (4). These uncertainties are partly due to the biochemical complexity of the plasma membrane and difficulties in measuring the molecular composition at the single-cell level with sufficient spatial resolution and in a noninvasive way.

Giant unilamellar vesicles (GUVs), consisting of a few selected phospholipid species and sterols, have been widely used as simplified models of cellular plasma membranes (5). In conditions that mimic biological processes, the GUV can separate into regions with different packing order. Regions characterized by a liquid-ordered (Lo) phase are typically enriched in saturated phospholipids and sterols, whereas regions in a liquid-disordered (Ld) phase consist mostly of unsaturated phospholipids (6). Curvature gradients can induce changes in packing order and spatial distribution of lipids. Thus, cyclical stretching of GUVs is a powerful method for investigating the interactions and distribution of phospholipids and cholesterol in plasma membrane. The role of cholesterol in stabilizing deformed lipid bilayers and its distribution between the two leaflets is still uncertain, despite it being the most abundant single molecule in the plasma membrane of animal cells (∼40 mol% of the total lipid in membrane) (7).

Curvature gradients in GUVs can be induced using a range of techniques, such as pulling tethers from adherent or pipette-aspirated GUVs (8, 9) or by stretching whole GUVs using optical tweezers (10, 11, 12, 13, 14, 15) and optical stretchers (16). Optical tweezers provide a flexible approach because they enable trapping and reversible stretching of individual GUVs manipulated within the field of view of an optical microscope to enable high-resolution imaging or molecular analysis. Previous studies using fluorescent-tagged phospholipids indicated that stretching GUVs can induce lateral separation of Lo and Ld phases, with Ld being predominant at the edges where the membrane curvature was higher (11). However, one limitation of fluorescence microscopy is the use of fluorescent labels (17, 18). The interactions of lipids within a bilayer is inherently dependent on their amphiphilic properties and molecular packing, both of which are potentially affected by fluorophores with sizes similar to the size of the lipid molecules. Recently, iSCAT, a label-free technique based on light scattering, was used to image phase-separated microdomains in supported lipid bilayers but did not provide molecular specificity (19). Neutron scattering (20) and mass spectrometry (21) can provide molecular specific information, but they are difficult to implement in configurations to allow simultaneous deformation and molecular analysis of individual GUVs. Raman microscopy has been used for imaging microdomains in supported monolayers (22). In addition, the use of stable-isotope-labeled lipids provides increased molecular specificity without adversely affecting the intermolecular interactions (22).

In combination with holographic optical tweezers (HOT), Raman microscopy enables trapping, manipulation, and molecular analysis of individual vesicles (23, 24, 25, 26). However, conventional HOT-Raman microscopes use a single (24) or multiple laser beams restricted to predetermined spatial patterns (27). This limits the ability to stretch individual GUVs and simultaneously measure Raman spectra at multiple membrane locations, corresponding to different curvature gradients. To overcome this limitation, we used a multibeam HOT-Raman instrument based on a liquid-crystal spatial light modulator (SLM) in the laser path and a digital micromirror device (DMD) in the Raman path. The synchronization of the SLM and DMD for excitation and detection allows an arbitrary array of sampling points from where Raman spectra can be simultaneously acquired (28), including for multiple optically trapped objects (29). Here, the integrated HOT-Raman microscope was used to measure the effect of curvature gradients on lipid ordering and cholesterol segregation in single GUVs. Optically trapped GUVs were stretched using the HOT, and multiple laser beams were used to measure Raman spectra simultaneously at the center (low curvature) and edges (high curvature) to investigate the effect of curvature gradients on the lipid sorting and packing. Deuterated cholesterol was used to increase the chemical specificity of the Raman readouts and provide spatially resolved and quantitative analysis of cholesterol concentration. A mathematical model was developed and provided information regarding the asymmetry of cholesterol concentration between the inner and outer leaflets of the bilayer.

## Materials and Methods

### Electroformation of GUVs

Samples of GUVs were prepared by the electroformation method originally described be Angelov and Dimitrov (30). The lipid reagents used were 1-palmitoyl-2-oleoyl-sn-glycero-3-phosphocholine (POPC, 850457; Avanti Polar Lipids, Alabaster, AL), cholesterol (Chol, ovine wool, 700000; Avanti Polar Lipids), sphingomyelin (SM, egg, 860061; Avanti Polar Lipids), and cholesterol-d6 (Chol-d6; 700172; Avanti Polar Lipids). Lipid mixes of 1:1:1 molar ratio were used for Chol/POPC/SM and Chol-d6/POPC/SM lipid blends. 10 mg of dry lipid powder was dissolved in chloroform. 0.2 mL of the chloroform/lipid solution was then pipetted onto an indium tin oxide (ITO)-coated glass slide (703192; Sigma-Aldrich, St. Louis, MO) with resistivity 8–12 *Ω*. The lipid-coated slide was left in a vacuum overnight to remove all traces of the solvent. To create the electroformation chamber, an o-ring was placed between the lipid-coated slide and a clean ITO-coated slide such that the conductive sides were facing. The chamber was filled with a sucrose solution (0.1 M) and sealed using binder clips. The ITO-coated slides were connected to a signal generator using crocodile clips and heated to 60°C. A square waveform was passed through the chamber at 1.2 Vpp and 10 Hz for 3 h. The voltage was then raised to 1.5 Vpp and the frequency reduced to 1 Hz and left for 1 h. The lipid vesicles were then collected from the chamber using a pipette and suspended in 1 mL of a glucose solution (0.1 M). Samples of vesicles were stored at −4°C and used within 24 h of preparation.

### Instrumentation: HOT-Raman microscope

**Figure 1:**
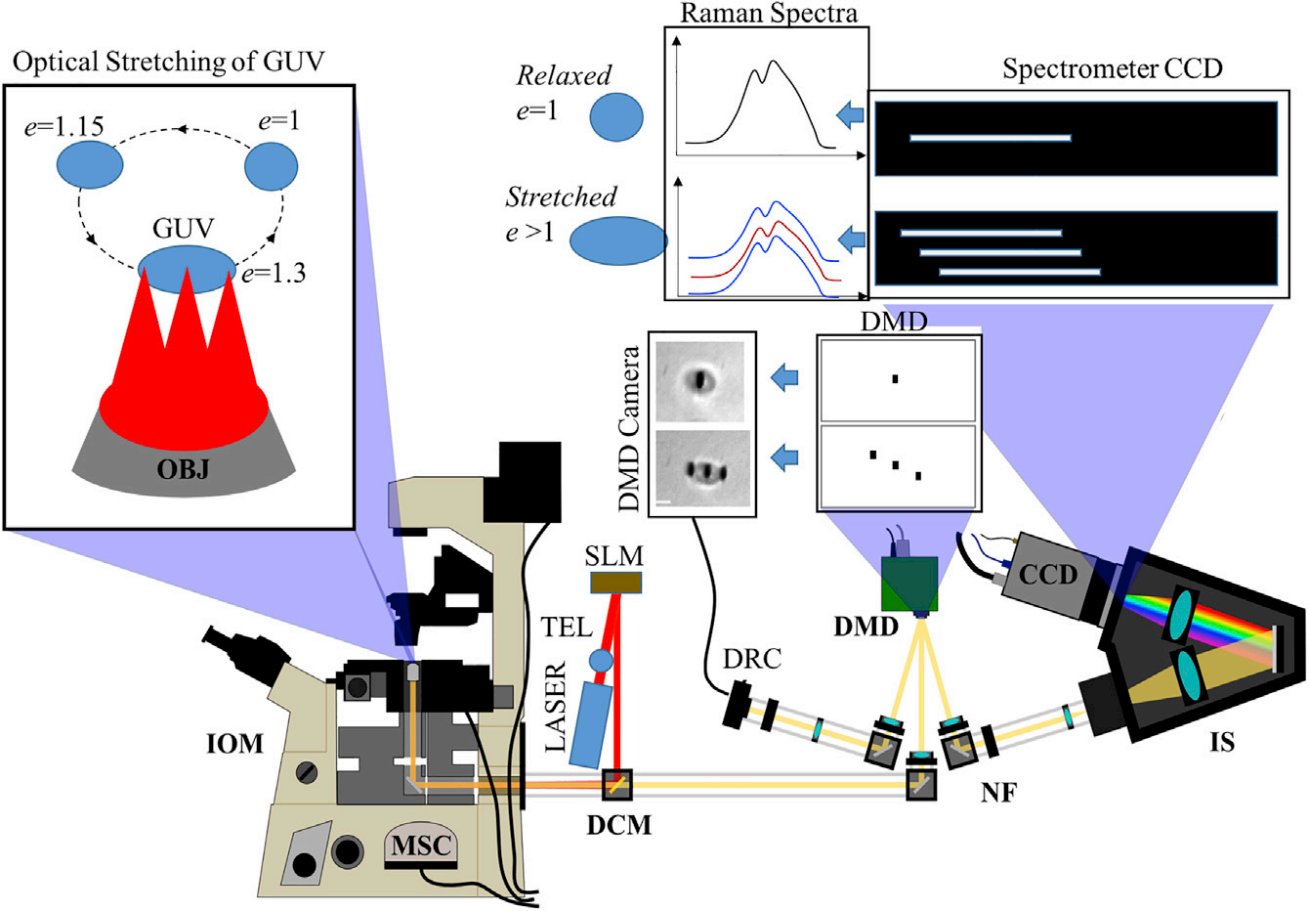
Schematic diagram of the integrated holographic optical tweezers (HOT) and Raman microscope. The instrument allows cyclical stretching of individual GUVs using three laser beams; the same laser beams are used to excite Raman measurements. For a vesicle, the elongation factor *e* is defined as the ratio between the long and short semiaxes. DCM, dichroic mirror; DMD, digital micromirror device; DRC, camera imaging the DMD; IOM, inverted optical microscope; IS, imaging spectrometer; MSC, microscope side-port camera; NF, notch filter; OBJ, microscope objective; SLM, spatial light modulator; TEL, telescope. To see this figure in color, go online.

A diagram of the HOT-Raman microscope is shown in Fig. 1 and has previously been described in (29, 31). A Ti:Sapphire laser (Spectra-Physics, Santa Clara, CA) tuned to 785 nm was used to form the optical tweezers (beam TEM_00_). Multiple laser foci were generated using a liquid-crystal SLM (BNS XY 512 phase with dielectric coating; Boulder Nonlinear Systems, Lafayette, CO/Meadowlark, Frederick, CO) controlled using the RedTweezers software (32). The position of each foci could be adjusted in three dimensions. The beam then overfills the back aperture of a 60×/1.2 NA water immersion objective (Olympus, Tokyo, Japan) to form multiple optical traps above a quartz slide (0.17 mm thick). The size of the laser spot, which defines the spatial resolution, was estimated using the diffraction limit to ∼800 nm, which ensures that three laser spots can probe the GUVs without overlap. Backscattered light was collected by the objective and reflected by a dichroic mirror (Semrock, Rochester, NY) toward a DMD (DLP Lightcrafter Evaluation module; Texas Instruments, Dallas, TX). The DMD pixels can be set so that they either reflect light toward an imaging camera (DCC1545M CMOS; Thorlabs, Newton, NJ) or toward the spectrograph (Acton LS785; Princeton Instruments, Trenton, NJ). The CMOS camera was calibrated (0.067 *μ*m/pixel) and was used to record bright-field images of GUVs and measure the size of the GUVs. A custom-made LabVIEW plugin has been written so that the coordinates set by the RedTweezers program are synchronized with adjustable oval-shaped reflecting elements on the DMD, reflecting Raman scattered light from the optically trapped particle onto the spectrograph. The spectrometer had a 3-mm-wide entrance slit and grating (1000 lines/mm) to enable acquisition of Raman spectra for a wide field of view while the DMD elements provided the spatial filtering (29). The detector used was a 128 × 1024 pixel CCD detector (Andor iDus BR-DD; Andor, Belfast, UK) cooled to −70°C.

### Optical stretching of vesicles

A suspension of lipid vesicles (50 *μ*L) was pipetted onto a quartz slide (thickness 0.17 mm) located on the software-controlled microscope stage. A vesicle was then optically trapped by adjusting the position of the stage. Raman spectra in the C-H and C-D stretching region (2000–3200 cm^−1^) were then recorded for the optically trapped vesicle. The size of the vesicles was measured by plotting the profile of the bright-field images along the elongation axis using ImageJ software. The diameter was measured as the distance between the local maxima at the edges of the GUV. Two additional traps were then positioned parallel to the initial trap on the edge of the vesicle using the SLM. The vesicle was then stretched by adjusting the x, y coordinates of the optical traps using the RedTweezers software controlling the SLM. Images of the vesicle and Raman spectra were recorded before and after the vesicle was stretched. The maximal elongation factor was found to be between 1.3 and 1.4. Any attempt to elongate further caused the vesicle to leave the optical trap. For vesicles containing deuterated cholesterol, the spectra were recorded with 60 s integration time; all other spectra were recorded with 30 s integration time. The vesicles were stretched using three optical traps (matched with oval-shaped reflective pinholes on the DMD), which were also the locations for the acquisition of Raman spectra. The polarization of the laser was set at ∼45° to the axis of elongation (confirmed by measuring Raman spectra of diphenylalanine microtubes with well-defined polarization-sensitive bands (33)).

### Data analysis

Raman spectra were recorded in the C-H and C-D stretching regions. For the C-H region, a linear baseline was fitted between 2750 and 3050 cm^−1^ and then subtracted. Spectra were then normalized to unity intensity at 2847 cm^−1^. The band intensities at 2847, 2882, and 2925 cm^−1^ were calculated as the average of the intensity at five CCD pixels nearest to the wavenumber. For the C-D region, a linear baseline was fitted by deconvolving the C-D region into a linear baseline and three Lorentzian peaks. The baseline was then subtracted from the spectra. The baseline-subtracted spectra were normalized to the intensity at 2847 cm^−1^ to avoid the difficulties related to intensity variations, which could be related to differences in lipid phase behavior at different positions. For vesicles containing deuterated cholesterol, the intensities *I*_*center*_ and *I*_*edge*_ were calculated as the sum of the three C-D bands *I*_*CD*_ = *I*_2132_ + *I*_2172_ + *I*_2243_. All spectra were smoothed using Savitzy-Golay (five data points, second-order polynomial).

## Results and Discussion

**Figure 2:**
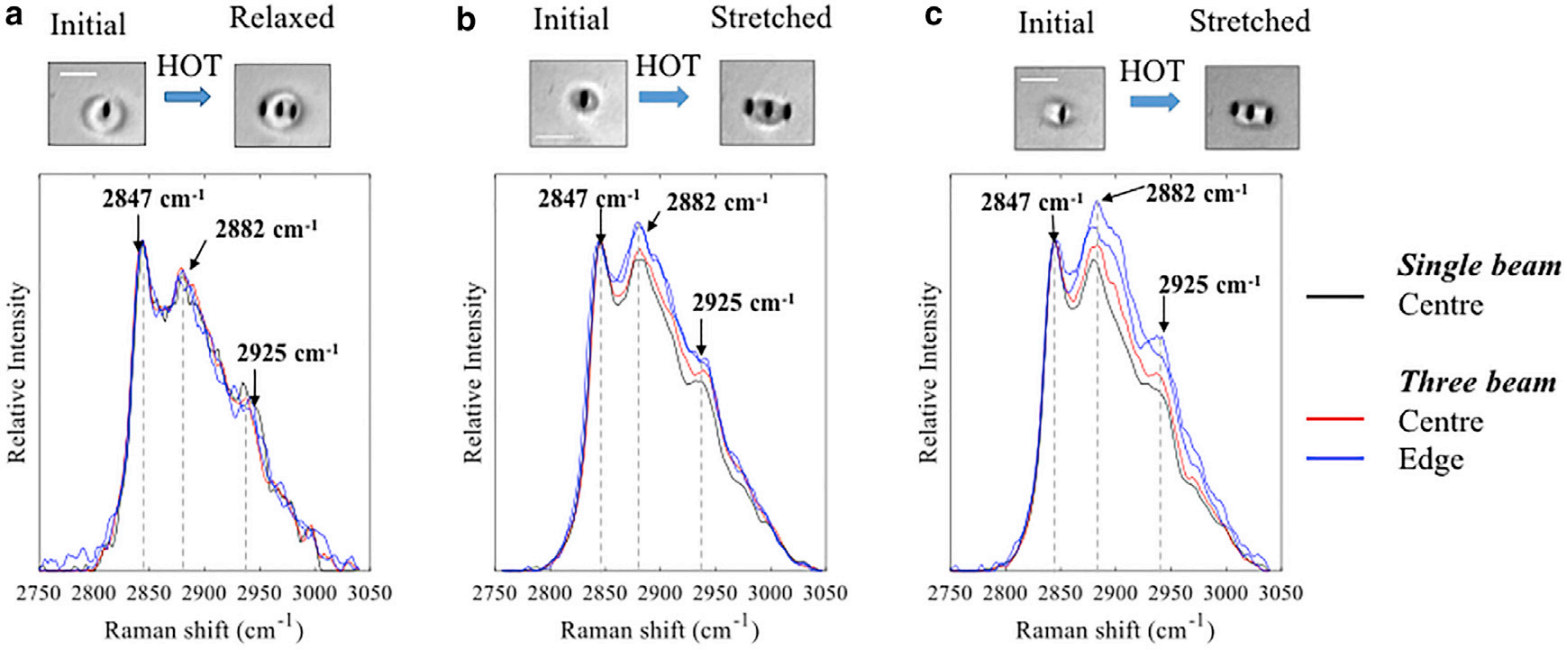
Spatially resolved Raman spectra of typical Chol/POPC/SM GUVs. (*a*) Relaxed vesicle and (*b* and *c*) two elongated vesicles stretched using the HOT are shown. The bright-field images (DMD camera) of the trapped GUVs, both in the relaxed and stretched state, show the positions of the laser beams for stretching and measuring Raman spectra. Scale bars, 5 *μ*m. To see this figure in color, go online.

First, we investigated the effect of optically stretching individual Chol/POPC/SM GUVs on the lipid chain packing. Fig. 2 compares the Raman spectra, recorded simultaneously, at the center and the edges of three typical GUVs, one relaxed (diameter 6.3 *μ*m) (Fig. 2 *a*) and two stretched (Fig. 2, *b* and *c*). The elongation factor of a GUV, *e*, was calculated as the ratio of the semiaxes. For the stretched GUVs, the initial diameters of the vesicles were 4.2 *μ*m (Fig. 2 *b*) and 5.3 *μ*m (Fig. 2 *c*), and were stretched to 5.5 *μ*m (elongation factor *e* ≈ 1.3) and 6.7 *μ*m (elongation factor *e* ≈ 1.28), respectively. The Raman bands are assigned to the symmetric methylene stretching *d*^+^ (2847 cm^−1^), antisymmetric methylene stretching *d*^−^ (2882 cm^−1^), Fermi resonance interaction between the symmetric methyl stretch and overtones for methylene bending *r*^+^ (2925 cm^−1^), and antisymmetric methyl stretch *r*^−^ (2959 cm^−1^) (34, 35).

Importantly, Fig. 2 *a* shows no spectral differences between the Raman spectra measured at the center and edges of relaxed GUVs. Considering that the lipid molecules have different orientations relative to the orientation of the laser polarization (before entering the microscope objective)—at the center, the molecules are predominantly oriented parallel to the axial direction, whereas at the edges, the molecules are predominantly perpendicular—the results show that the experimental conditions provide Raman spectra insensitive to the orientation of the molecules. This is important to ensure that any spectral changes observed during stretching of GUVs can be assigned to lipid packing behavior rather than changes in molecular orientation. Sensitivity to molecular orientation has been previously reported when polarized Raman spectroscopy was used to study lipid monolayers (36). This study employed a microscope objective with low NA = 0.4 and a cross-polarized configuration (polarizer in the path of the Raman scattered light). This is different from our instrument, in which the objective had NA = 1.2 (high NA objectives alter the polarization of the laser by creating a strong component in the axial direction) and no polarizer in the Raman path, and the in-plane component of the laser polarization was aligned at a 45° angle to the GUV elongation axis. Fig. 2 *a* confirms that the experimental conditions are not sensitive to the orientation of the molecules, parallel or perpendicular to the axial direction.

For lipid bilayers, the height ratios of the *d*^+^ and *d*^−^ bands (*I*_2847_/*I*_2882_) and *d*^+^ and *r*^+^ bands (*I*_2847_/*I*_2925_) are well-known to be sensitive to the packing order of the lipid tails, although a precise interpretation was found to depend on the lipid blend of the membrane. In pure phospholipid bilayers, an increase in packing order was shown to be accompanied by an increase in the ratio *I*_d+_/*I*_d−_ (*I*_2847_/*I*_2882_) (37, 38). However, for Chol/POPC/SM vesicles, an opposite effect was observed: the ratio *I*_d+_/*I*_d−_ decreased when lipid chain ordering was decreased by increasing the temperature of individual vesicles from 20 to 60°C (24).

The results in Fig. 2 show that the optical stretching of the Chol/POPC/SM GUVs leads to a decrease in both *I*_d+_/*I*_d−_ and *I*_d+_/*I*_r+_ ratios in the Raman spectra measured at the edges compared to the initial spectra. These results indicate that stretching leads to a decrease in lipid chain packing at the edges of the GUVs compared to the center. Although measuring the Raman spectra at the left and right edges of the GUVs at equal distances to the central position was tried, this was not always achieved. The limited accuracy for setting the coordinates of the traps represents the main experiment uncertainties. The results in Fig. 2 *b* correspond to an experiment in which a high level of symmetry was achieved, leading to almost identical Raman spectra at the edges and only small differences at the center before and after stretching the GUV. However, for the GUV in Fig. 2 *c*, the distances from the spectral acquisition points at the left and right edges to the center are visibly different. The Raman spectrum measured at the right edge spot, which is closer to the central spot, has higher *I*_d+_/*I*_d−_ and *I*_d+_/*I*_r+_ ratios compared to the spectrum acquired at the left edge spot, which is further away from the center but closer to the GUV edge. The results obtained in these “asymmetric” conditions suggest that the packing order decreases with increasing distance from the center.

**Figure 3:**
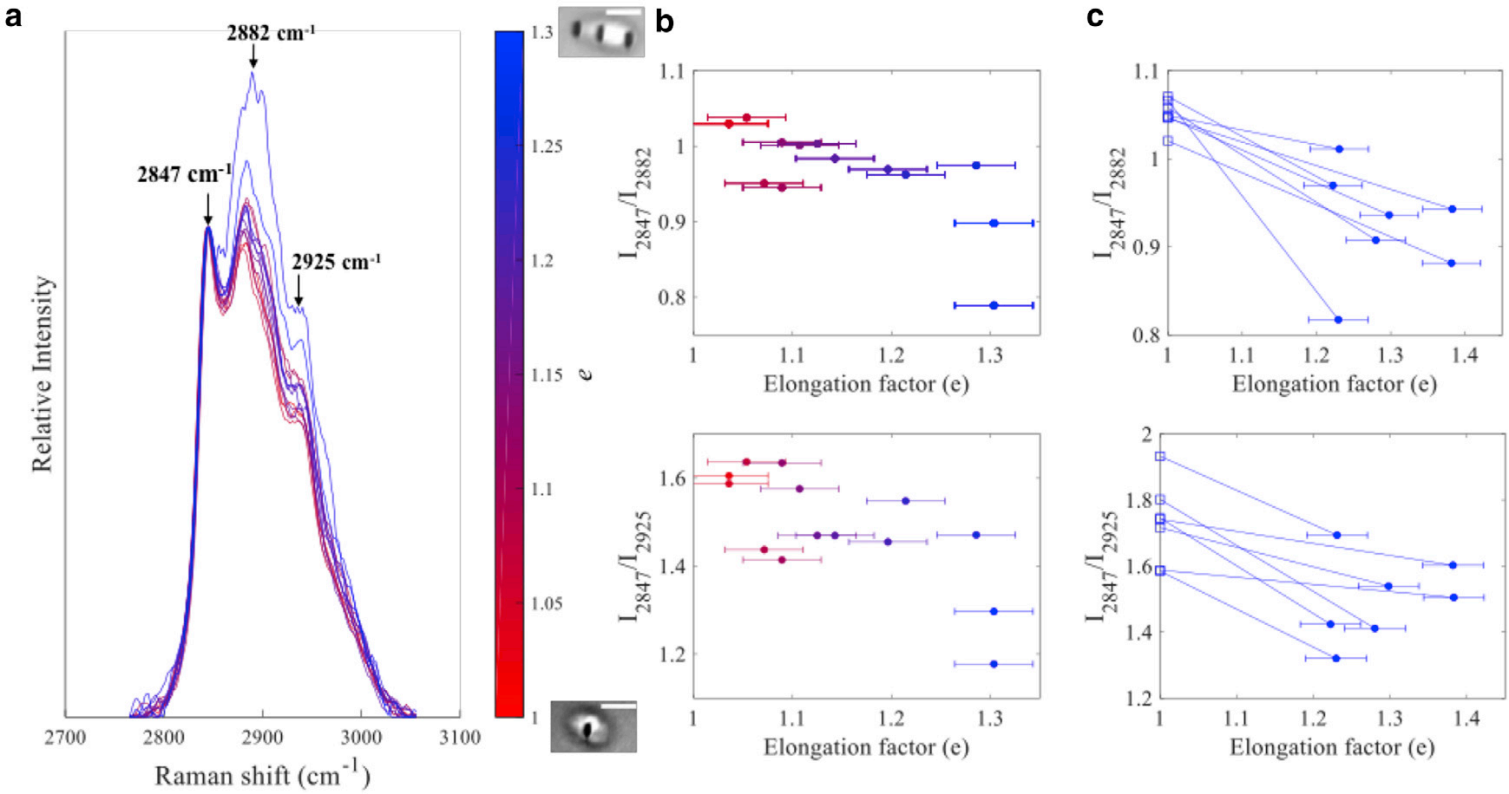
(*a*) Raman spectra measured at the edges of a typical Chol/POCP/SM GUV cyclically stretched using HOT. Spectra are normalized using the intensity of the *d*^+^ band (2847 cm^−1^). (*b*) Calculated intensity ratios I_2847_/I_2882_ and I_2847_/I_2925_ at different elongation factors for the GUV in (*a*) are shown. (*c*) Intensity ratios I_2847_/I_2882_ and I_2847_/I_2925_ calculated from Raman spectra of additional seven GUVs are shown. Each guiding line corresponds to individual GUVs. Horizontal error bars represent uncertainties in identifying the coordinates of the GUV edges (the *vertical error bars* are negligible). To see this figure in color, go online.

To quantify the effect of membrane curvature gradients on the relative intensity of the C-H stretching bands, individual vesicles were stretched to a range of elongation factors *e* between 1 and 1.3. Fig. 3 *a* shows 14 Raman spectra measured at the edges of a single Chol/POCP/SM GUV cyclically stretched within this range of elongation factors. Increasing the elongation factor led to an increase in the intensity of the *d*^−^ and *r*^+^ bands relative to the *d*^+^. The calculated values of the *I*_2847_/*I*_2882_ and *I*_2847_/*I*_2925_ ratios from the Raman spectra in Fig. 3 *a* are presented in Fig. 3 *b*. Because the spectra taken from the right and left spots may differ because of the symmetry of the laser spots relative to the center of the vesicle, the spectrum with the minimal value of the *I*_2847_/*I*_2882_ was selected because it was more likely to be closer to the edge. The results in Fig. 3 *b* show that the intensity ratios *I*_2847_/*I*_2882_ and *I*_2847_/*I*_2925_ decreased from 1.03 and 1.6 at the lowest elongation factor (*e* ≈ 1.01) to 0.79 and 1.18 at the maximal achievable elongation factor for our experiments (*e* ≈ 1.3). The findings were confirmed by measurements on seven more GUVs, for which Raman spectra were collected at two elongation points (Fig. 3 *c*).

These results provide conclusive evidence of significant changes in lipid chain packing upon optical deformation. Although the stretching induced changes differ among vesicles, the effects are clearly seen in the spectra acquired at the single-GUV level (reduces uncertainties caused by small differences in size or lipid composition between GUVs). For Chol/POPC/SM bilayers, phase-separated Lo and Ld domains coexist in the GUV in the relaxed state, but they have different capabilities to curve (2, 39). Deforming the GUV by optical stretching leads to curvature gradients across the bilayer, which can induce sorting of individual lipids based on the molecular shape (40) as well as lateral segregation of Ld and Lo microdomains (41). Our results show that the Lo phase is predominantly found at regions with low curvature (center of GUV), likely because the Lo phase membranes show a higher resistance toward bending compared to Ld phase domains. Our results are in agreement with previous experiments using GUVs containing rhodamine-dihexadecanoyl phosphoethanolamine that highlighted an Ld phase at the edges of optically stretched GUVs (11). Similar curvature-induced sorting was also observed in membrane tubes pulled from GUVs, where the fluorescent labels used for the Ld domain were found in higher concentration in the high-curvature tubes (nanoscale) compared to the main GUV (low curvature) (42). However, the main advantage of Raman spectroscopy is that no fluorescent labels are required to observe and quantify the curvature-induced changes in lipid packing.

Next, we investigated the effects of curvature gradients on lipid sorting by measuring quantitative and spatially resolved information regarding the abundance of different lipid types within the bilayer. Because the C-H stretching region of the Raman spectrum contains contributions from all lipids, it is difficult to discriminate between different types of lipids, although more complex statistical methods have been shown to be able to overcome this for the fingerprint (43) and C-H region (44). Therefore, we used stable isotope labeling to isolate the Raman bands corresponding to the labeled molecular species. Unlike the large probes used in fluorescence microscopy, stable isotope probes used in Raman spectroscopy are unlikely to alter physical interactions governing bilayer organization. Here, natural cholesterol was substituted for Chol-d6, in which six hydrogen atoms have been replaced with deuterium. This substitution induces new C-D stretching bands in the Raman spectrum that can be identified in the previously silent region 2000–2300 cm^−1^.

**Figure 4:**
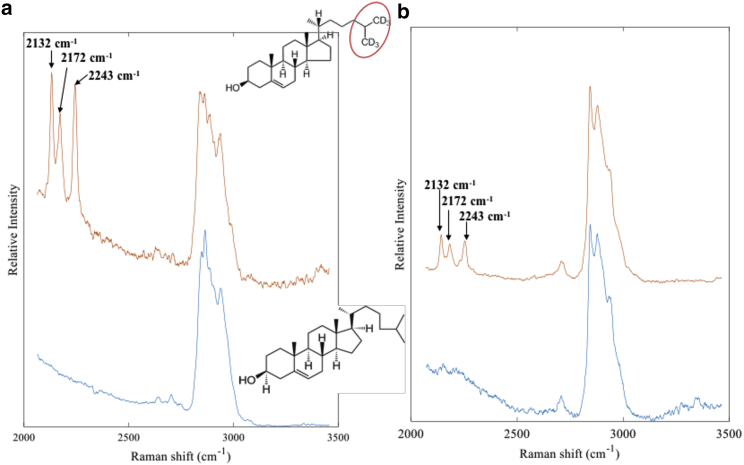
(*a*) Raman spectra of cholesterol and deuterated cholesterol (Chol-d6) (dry powder). (*b*) A comparison between Raman spectra of relaxed Chol-d6/POPC/SM and Chol/POPC/SM GUVs is shown. To see this figure in color, go online.

Fig. 4 compares Raman spectra of cholesterol and Chol-d6 and Chol/POPC/SM and Chol-d6/POPC/SM GUVs. For Chol-d6, three Raman bands corresponding to the C-D stretching vibrations can be identified at 2132, 2172, and 2243 cm^−1^. Fig. 4 *b* shows that Chol-d6 can be detected with high sensitivity even at the single-GUV level. Apart from the C-D bands, no other spectral differences were observed in the C-H stretching region, confirming that the bilayer structure and packing were not altered by deuterium substitution.

**Figure 5:**
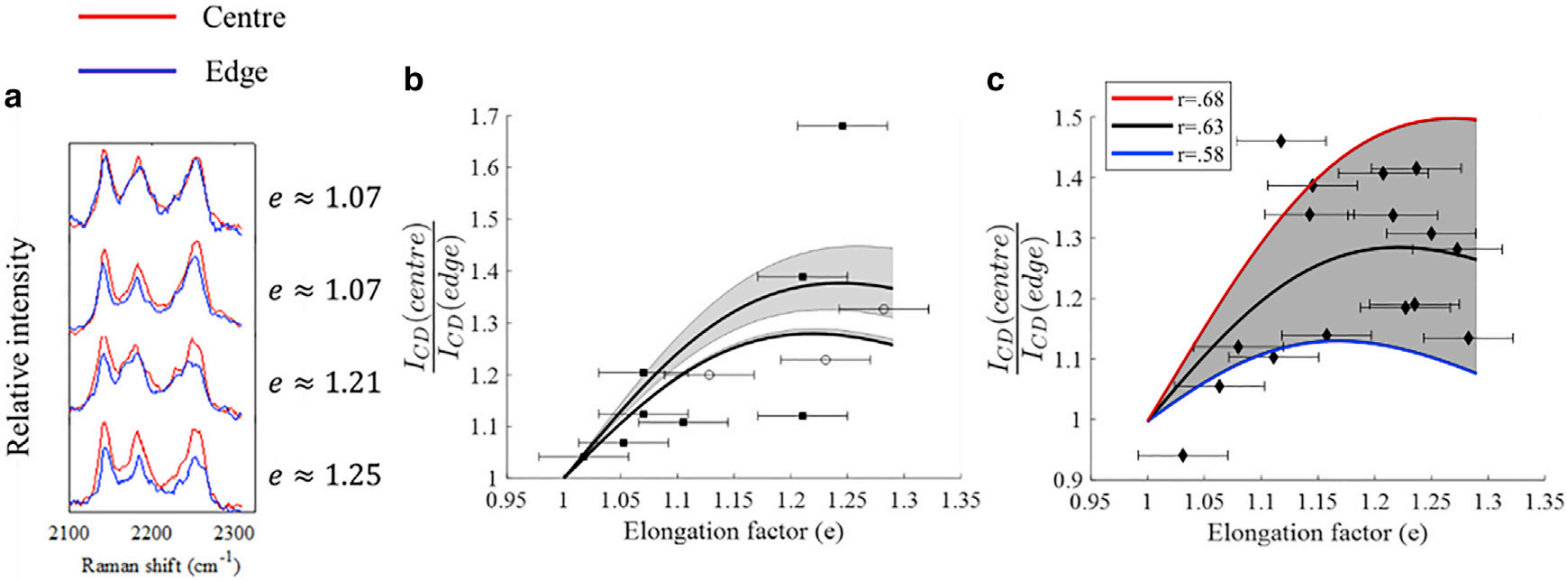
(*a*) Spatially resolved Raman spectra for a typical Chol-d6/POPC/SM GUV at different elongation factors showing the C-D bands. Spectra are vertically offset for clarity. (*b*) Fit of the ratio of the C-D band intensities measured at the center and edge of a vesicle *I*_*CD*_(*center*)/*I*_*CD*_(*edge*) to the theoretical model (Eqs. 1, 2, and 3) is shown for two GUVs (data points indicated by *black squares* and *white circles*). (*c*) Fit of the band intensity ratio *I*_*CD*_(*center*)/*I*_*CD*_(*edge*) to the theoretical model for data obtained from 17 GUVs is shown, yielding a mean value *r* = 0.63. The lines corresponding to the ±0.05 error range (*r* = 0.68 and *r* = 0.58) are also included. Error bars indicate estimated experimental uncertainties in determining *e*. To see this figure in color, go online.

To investigate whether the curvature-induced changes in lipid packing are accompanied by lateral segregation of Chol-d6, individual Chol-d6/POPC/SM GUVs were optically stretched, and Raman spectra were measured at the center and at the edges of the vesicles. Fig. 5 *a* presents examples of typical Chol-d6/POPC/SM GUVs, stretched as several elongation factors. The Raman spectra in Fig. 5 show that increasing the elongation of the GUVs leads to a decrease in the intensity of the C-D stretching Raman bands assigned to Chol-d6 at 2132, 2172, and 2243 cm^−1^. These spectral changes indicate that the decrease in lipid chain ordering at the edges of the vesicle, which have a higher curvature, is associated with a lower cholesterol concentration. The curvature gradients induced by the stretching of the bilayer lead to an overall lateral segregation of cholesterol and/or cholesterol-rich Lo domains toward the lower-curvature regions at the center of the vesicle.

It is important to note that the Raman spectroscopy results provide an overall estimation of the cholesterol concentration (proportional to the intensity of the C-D bands) for both leaflets of the bilayer. However, because the curvature in the two leaflets have opposite signs, the segregation of Chol in the two leaflets will be in opposite directions. To provide a better understanding of the spatial distribution of cholesterol in the bilayer, a previously proposed mathematical model (45) was used to compare the predicted spatial distribution of cholesterol in a stretched GUV with the Chol-d6 concentration measured from the Raman spectra (concentration is proportional to the intensity of the C-D stretching bands). Because the curvature-induced segregation of Chol is different in the two leaflets, the model includes both leaflets and considers an asymmetry in cholesterol concentration between the inner and outer leaflets. The cholesterol asymmetry is still a controversial topic (7), and a range of values have been reported for membranes of different lipid compositions. To our knowledge, no measurements of the interleaflet asymmetry of cholesterol has been reported for the Chol/POPC/SM blend used in this study.

Although translocation of cholesterol from one bilayer leaflet to the other (flip-flop exchange) can occur in phospholipid membranes at rates as high as 10^4^ s^−1^ (46), higher energy barriers were found for lipid blends containing phase-separated Lo and Ld domains (47). For the Chol/POPC/SM membrane used in this study, the flip-flop rate was estimated to be 30 min (48). Considering the lack of data regarding the equilibrium distribution of cholesterol sterols across the bilayer and the relative short timescales of our experiments (few minutes), the interleaflet flip-flop exchange was not included in the model for simplicity.

According to the spontaneous curvature model developed by Tian et al. (45), the expressions for cholesterol concentration profile in the outer (*K*_*o*_) and inner (*K*_*i*_) leaflets of a bilayer optically stretched are

(1) $$K_{o}=\exp\left(\frac{k_{c}aHC}{k_{B}T}\right),\;\;\;\;\;\;\;\;\;\;\;\;\;\;K_{i}=\exp\left(-\frac{k_{c}aHC}{k_{B}T}\right),$$

where *k*_*c*_ is the bending stiffness, *a* is the molecular cross-sectional area, *H* is the mean curvature, *C* is the spontaneous curvature, *T* is the absolute temperature, and *k*_*B*_ is Boltzmann constant. The optically stretched vesicle can be modeled as an ellipsoid elongated by a factor of *e* in the *z* direction. In parametric form, the ellipsoid is given by $\left[\left(u,v\right),\;\;y\left(u,v\right),\;\;z\left(u,v\right)\right]=\frac{d}{2}\left[\cos\left(u\right)\sin\left(v\right),\sin\left(u\right)\sin\left(v\right),e\;\cos\left(v\right)\right]$, where *d* is the diameter of the relaxed vesicle and *u* and *v* are spherical coordinates. The above parameterization gives the following expression for mean curvature:

(2) $$H=\frac{e\left(3+e^{2}+\left(2-2e^{2}\right)\cos\left(2v\right)\right)}{2d{\left(\cos^{2}\left(v\right)+e^{2}\;\sin^{2}\left(v\right)\right)}^{\frac{3}{2}}}.$$

Therefore, the intensity of the C-D Raman bands *I*_*CD*_ is proportional to the total concentration of cholesterol in the membrane, which at a position away from the center of the vesicle may be expressed as

(3) $$I_{CD}\propto K=rK_{o}+\left(1-r\right)K_{i},$$

where *r* is a parameter describing the asymmetry of cholesterol in the two leaflets. Considering that values for *k*_*c*_, *a*, and *C* have been reported with good agreement in the literature (*k*_*c*_ = 86.8 *k*_*b*_*T*, *a* = 0.265*/*nm^2^, and *C* = −0.494 1/nm) (49, 50, 51), we first compared the model to the intensity of C-D bands, *I*_*CD*_, obtained from individual Chol-d6/POPC/SM GUVs stretched cyclically to different elongation factors. Fig. 5 *b* presents the calculated ratio of the C-D band intensities at the center and edges *I*_*CD*_(*center*)/*I*_*CD*_(*edge*) for two typical GUVs.

**Figure 6:**
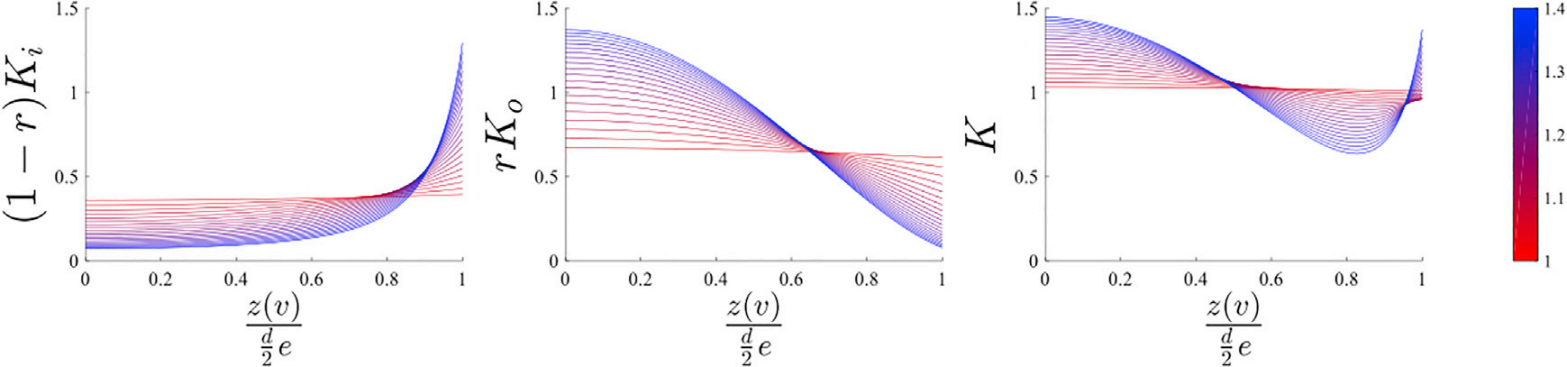
Theoretical estimation of the cholesterol concentration profiles in the inner ((1 − *r*)*K*_*i*_) and outer (*rK*_*o*_) leaflets and the overall bilayer (*K*) induced in the optically stretched GUVs. The results are obtained from Eqs. 1 and 2 using a mean *r* = 0.63 from Fig. 5. To see this figure in color, go online.

Because the intensity *I*_*CD*_ is proportional to the total concentration of cholesterol *K*, the asymmetry parameter *r* can be estimated by fitting the intensity of the Raman bands to the theoretical model (Eqs. 1, 2, and 3) using *r* as the fitting parameter. Because Raman spectra were acquired only at the center and edges of the GUVs, *r* was considered uniform to avoid overfitting. Except for a few outlier values, Fig. 5 *b* shows that the ratio *I*_*CD*_(*center*)/*I*_*CD*_(*edge*) for the two vesicles follows the trend of the model, providing very similar values for the asymmetry factors: *r* = 0.65 ± (0.02/0.01), *r* = 0.628 ± (0.002/0.001) (the uncertainty was estimated by running the model using extreme values for the measurement of elongation factor). Although we do not have a clear understanding of the outlier values, interleaflet translocation of cholesterol, which was neglected in our model, may be one reason. Close inspection of the Raman data upon cyclical stretching of the GUVs did not reveal any hysteresis. Fig. 5 *c* presents further results obtained from on 17 more GUVs to account for possible heterogeneity in membrane blend and sizes. The model fit to the *I*_*CD*_(*center*)/*I*_*CD*_(*edge*) data from all GUVs provided *r* = 0.632 ± (0.005/0.002), which is consistent with the values obtained for the single-GUV cyclical stretch in Fig. 6 *b*. Using a model that considers the increase in surface area of the vesicle upon stretching (equivalent to an increase of *a* by ∼20% at *e* = 1.3) leads to a lower value of *r* = 0.629 (when fitting the data for all vesicles shown in Fig. 5 *c*).

These results provide evidence that the outer leaflet of the bilayer has significantly higher cholesterol content than the inner leaflet. Although no value of *r* has been reported in the literature for Chol-d6/POPC/SM membranes with 1:1:1 relative concentration, for cells, the literature reports a broad range of values of *r* ranging from 0.20 to 0.90 (7). The wide range of *r* is partly caused by uncertainties related to the analytical techniques used, which often required destructive measurements or insertion of bulky fluorescent labels. Theoretical models for artificial lipid bilayers indicated that cholesterol asymmetry depends on the lipid composition, and in a membrane consisting of a bilayer with asymmetric phospholipid composition and cholesterol, the cholesterol is predicted to have higher concentrations in the inner leaflet, which contained higher amounts of phosphatidylserine (52, 53, 54). Nevertheless, a model consisting of 63 species of lipids (different head and tail sizes) reported a cholesterol distribution favoring the outer over the inner leaflet by 5446% (55). Simulations have also predicted an asymmetry favoring the outer leaflet of 64% (56).

Using the value *r* = 0.63 (mean for the 17 GUVs analyzed here), the theoretical model was used to calculate the distribution of Chol-d6 in stretched GUVs at various elongation factors, and thus provide an indication for the possible dynamics of cholesterol during the deformation. Fig. 6 shows that the curvature gradients in the membrane induce large lateral movement of Chol in both inner and outer leaflets to minimize the free energy required for the high curvature at the edges. The calculated cholesterol concentration was plotted against the position along the axis of elongation relative to the size of the vesicle and elongation factor (the concentration was normalized so that when *e* = 1, the cholesterol concentration is 1 across the vesicle). The results suggest that the spatial changes in cholesterol distribution lead to changes in lipid packing across the GUV: high ordering at the center and lower ordering at the edges, as indicated by the relative intensities of the C-H stretching bands. For the outer leaflet, given the positive spontaneous curvature of the phospholipids and the negative spontaneous curvature of Chol, the curvature gradient induces a lateral movement of cholesterol toward the low-curvature region. This segregation in the outer leaflet is accompanied by lateral movement in the inner leaflet but in an opposite direction, from the center to the edges. Increasing the elongation, the segregation in both leaflets are predicted to increase. At the maximal elongation factors of *e* = 1.3, the model predicts that the Chol concentration at the edges is fivefold lower than at the center for the outer leaflet and ∼7-fold higher in the inner leaflet, suggesting large local interleaflet asymmetry. However, the measured Raman spectra capture the overall concentration of the Chol in the membrane and cannot discriminate between the two leaflets. The model predicts that, overall, at an elongation factor of ∼1.3 (maximal achievable in these experiments), the concentration of Chol is ∼1.3-fold higher at the center compared to the edges, which is in agreement with our experimental findings. It is interesting to note that the model predicts a minimum in the total concentration of Chol when $\left(z\left(v\right)/\frac{d}{2}e\right)$ = 0.83. However, verifying this prediction was not possible with the current instrument, which does not provide the required spatial resolution to measure Raman spectra from five closely spaced positions.

## Conclusions

The link between curvature gradients in a lipid bilayer and lipid chain packing and cholesterol distribution was investigated using an integrated optical tweezers Raman microscope. The instrument allowed multiple Raman spectra to be simultaneously acquired from different regions of a cell-sized, optically stretched lipid vesicle. Cyclic stretching of a single vesicle resulted in a gradual change in the intensity profile of the C-H stretching region. This was interpreted to correspond to a decrease in ordering at the edge dependent on curvature gradient of the vesicle. To quantify the difference in cholesterol concentration along the vesicle, cholesterol was substituted for deuterium-labeled cholesterol (Chol-d6). The relative intensity of the C-D bands was used to measure the difference in cholesterol concentration between edge and center. These data were fitted to a mathematical model based on spontaneous-curvature-based sorting and an asymmetry between cholesterol concentration in each leaflet. The model predicts a value *r* = 0.63 describing the asymmetry in cholesterol concentration between the bilayer leaflets. The results demonstrate the potential of the integrated HOT-Raman technique to induce deformations to individual lipid vesicles and to simultaneously provide quantitative and spatially resolved molecular information. Future studies can extend to include more realistic models of cell membranes (artificial lipid- or cell-derived membranes) and, potentially, live cells.

## Author Contributions

I.N. and L.C. conceived the project, designed experiments, and wrote the manuscript. L.C. carried out experiments and analyzed data. F.S. developed the instrumentation.
